# Examining Learning Resources Used by Year 5 Medical Students for UKMLA Preparation: A Descriptive Case Study

**DOI:** 10.1007/s40670-026-02665-z

**Published:** 2026-02-25

**Authors:** Soban Sadiq, Manfred Gschwandtner, Zachary Hollenberg, Claire Parkin

**Affiliations:** Kent and Medway Medical School, University of Kent, Canterbury, UK

**Keywords:** UKMLA, Learning resources, Medical students, AKT, CPSA, Exam preparation

## Abstract

**Introduction:**

The UK Medical Licensing Assessment (UKMLA), introduced in 2024/25, is a national requirement for all final-year UK medical students. This Case Study explores how Year 5 students at Kent and Medway Medical School (KMMS) prepared for both components of the UKMLA, the Applied Knowledge Test (AKT) and the Clinical and Professional Skills Assessment (CPSA).

**Methods:**

An electronic survey containing Likert-scale, multiple-choice, and open-text questions was distributed to the Year 5 cohort (*n* = 64) after the AKT and before the CPSA. Thirty-one students (48%) responded. Resource use was ranked using weighted scores based on frequency. Fisher’s Exact Test was used to explore associations between demographic variables and resource preferences.

**Results:**

Students relied heavily on third-party resources for AKT preparation, with *PassMedicine* achieving the highest weighted score (121). Other commonly used tools included *Anki*,* Geeky Medics*, and *Zero to Finals*. KMMS in-house materials were more commonly used for CPSA preparation. On average, students spent 80% of their preparation time using external resources. Demographic analysis showed older students more frequently used *Essential Primary Care* (*p* = .016) and *Pastest* (*p* = .030). Female students used internet-based resources more than males (*p* = .043), while younger students favoured *Geeky Medics Flashcards* (*p* = .041). *ChatGPT* was also used for explanations and personalised support.

**Conclusion:**

Third-party tools dominate AKT preparation. Age and gender influence resource choices, and AI-based tools are emerging as study aids. To promote equitable access, medical schools should consider providing key third-party resources. Further research should evaluate how resource combinations impact UKMLA performance.

## Introduction

The UK Medical Licensing Assessment (UKMLA) is a national exam taken by every final year medical student since the academic year 2024/2025 [1]. Alongside their medical degree, passing this national exam is essential for joining the medical register. The UKMLA has two components; one is a written assessment of applied clinical knowledge, known as the Applied Knowledge Test (AKT) and the other is a performance-based assessment, known as the Clinical and Professional Skills Assessment (CPSA) [1]. Kent and Medway Medical School (KMMS) is a recently accredited medical school which opened to its first cohort in 2020. In 2025, the first cohort, now Year 5 medical students, were the first to undertake their UKMLA at KMMS.

The aim of this study was to conduct a descriptive case study examining the learning resources used by KMMS Year 5 (Y5) students, in preparation for both parts of the UKMLA. As the UKMLA was undertaken by all UK medical students for the first time in the academic year 2024/25, research literature on this topic is scarce. To our knowledge, no previous study has interrogated the resources that students are using to prepare for their UKMLA.

Alongside the UKMLA, there are a range of other national licensing examinations such as the United States Medical Licensing Examination (USMLE), the Medical Council of Canada Qualifying Examination (MCCQE), the German Staatsexamen, the Swiss Federal Licensing Examination (FLE) and the Japanese National Medical Licensing Examination (NMLE) [2]. With the exception of the USMLE, no literature was found for any of the other licensing examinations, therefore, the following background information draws exclusively on USMLE literature.

Third-party resources are commercial learning resources that are specifically designed for exam preparation [3–5] .They include questions banks, flashcards, video platforms or high yield exam preparation books such as the *First Aid for the USMLE Step1* [6] These resources have become the dominant learning resources in preparation for the USMLE [3, 4, 7]. They eclipse the use of traditional textbooks, and any school material provided for the USMLE [8]. Amongst third-party resources question banks hhave emerged as the most frequently used resources for exam preparation [9–11], with further studies showing that their use is positively correlated with performance in USMLE exams [7, 12–14]. The main reasons for using question banks are easy accessibility and usage, simulated exam experience, identifying gaps in knowledge and to consolidate exam preparation [11]. In addition, completing a high number of questions can help reduce stress and anxiety as it gives students a feeling of preparedness [11]. Alongside question banks, *Anki* flashcards and video platforms like *Pathoma* or *Osmosis* have also emerged as a popular resource for USMLE preparation [3, 9, 14]. *Anki* uses spaced retrieval practice to improve long term retention [15] and their use is associated with superior performance in USMLE exams [3, 14, 16]. Whilst third-party tools are most frequently used by medical students to prepare for the USMLE, traditional textbooks still form part of the resources mix they use. Compared to high yield exam preparation books, textbooks provide an authoritative and comprehensive in-depth synthesis of a specific medical subject [17]. Although they are used less by students [3, 8] a study by Ranabhat et al. [7] found a significant correlation between traditional textbooks and student performance in the USMLE basic science stage. These results suggest that textbooks are still relevant for USMLE preparation. Third-party tools are often not provided by medical schools which can lead to additional financial burden for students [18] and, as a consequence, to inequalities where some students might not be able to afford them [3]. Because of this, and the clear indication that third-party tools improve performance on national licensing exams such as the USMLE, medical schools should consider integrating these resources into their course and discussing their benefits and disadvantages with students [3, 11].

KMMS provides its students with a range of third-party resources including question banks (*Passmedicine*, Clinical Key Students Question bank), MLA Mock exams, review books such as the Crash Course and At a Glance series, a video platform (*Osmosis)*, a bite-sized learning platform (*RX Bricks*) and a case-based learning app (*Capsule*). In addition, the school offers access to traditional textbooks such as *Kumar and Clark’s Clinical Medicine* or *Macleod’s Clinical Examination* and to a range of resources produced in-house, such as lecture slides or skills worksheets.

The aim of this study was to conduct a descriptive case study examining the learning resources used by Year 5 students at KMMS, in preparation for the United Kingdom Medical Licensing Assessment (UKMLA) Applied Knowledge Test (AKT) and Clinical and Professional Skill Assessment (CPSA).

In order to achieve this, the study:

- investigated the frequency with which students utilised external resources (third-party tools and textbooks) and the schools’ in-house resources to prepare for the AKT and the CPSA component.
- explored the motivation underlying students’ use of these resources.
- compared resources funded and provided by the school with resources currently not provided.
- examined how much time students engaged with the schools’ in-house (KMMS-produced) resources compared to external resources.

These insights may be beneficial to other UK medical schools in terms of student UKMLA preparation support, as well as to evidence business cases for purchase of third-party resources. Furthermore, they will help to better understand the usefulness of in-house resources produced by the medical school.

## Methods

### Study Design

This was a descriptive case study, using an eSurvey. It was conducted in April 2025, following the Year 5 students’ sitting of the UKMLA-AKT, but before they undertook their UKMLA Clinical and Professional Skill Assessment (CPSA). The survey was administered between the two UKMLA assessments, because more responses were expected than after the CPSA, when students would have moved on to a different part of the curriculum.

### Participants

Using a purposive sampling approach, all final year medical students at KMMS were invited to participate in the study. For 95% confidence level, with 5% margin of error, the representative sample size required was 55 students, out of the total Year 5 cohort size of 64 students. Recruitment was undertaken via student newsletter, the Year 5 students’ WhatsApp group and during an end of module feedback forum. Recruitment ran across 5 weeks.

### Ethical Approval

Participant information and consent details were embedded within the eSurvey. Consent was implied, based on participant willingness to complete and submit their survey response. All responses were anonymous, to help mitigate against confirmation bias. Ethical approval for this study was obtained from the KMMS Research Ethics Advisory Group (REAG): Approval ID: 2,503,038.

### Data Collection

The eSurvey, constructed within the Qualtrics.^XM^ survey platform contained a mix of multiple choice and open questions. Demographic questions focussed on age, gender, level of study and if participants have previously worked as registered health professionals. Some of the Multiple-Choice Questions used a Likert Scale (MCQ-LS) to understand the *frequency* of use of specific resources. Open answer (free text) questions, as well as some other MCQs with Predefined Answer Responses (MCQ-PAR), enabled students to describe *why* they were using these resources (Table 1).

**Table 1:** eSurvey questions

eSurvey Questions	Question type
Resources for preparing the MLA AKT Exam	
Books	
1. How often did you use the following **books** from the Y5 Senior Rotations Reading list to prepare for the MLA AKT exam?	MCQ-LS
2. Are there any other books that you used **often** which are not mentioned in this list?	Free text
3. How did textbooks (e.g. Kumar and Clark’s Clinical Medicine) help you to prepare for the MLA AKT exam?	MCQ-PAR
Question Banks	
4. How often did you use the following **question banks** to prepare for the MLA AKT exam?	MCQ-LS
5. Why did question banks help you to prepare or the MLA AKT exam?	MCQ-PAR
Flashcards	
6. How often did you use the following **flashcard** platforms to prepare for your MLA AKT exam?	MCQ-LS
7. Why did Flashcards help you to prepare for the MLA AKT exam?	MCQ-PAR
Learning Platforms	
8. How often did you use the following **learning platforms** to prepare for the MLA AKT exam?	MCQ-LS
9. How did these learning platforms help you to prepare for the MLA AKT exam?	MCQ-PAR
Clinical Decision Support Tools (e.g. UpToDate)	
10. How often did you use the following **clinical decision support tools** to prepare for your MLA AKT exam?	MCQ-LS
11. How did these clinical decision support tools help you to prepare for the MLA AKT exam?	MCQ-PAR
Artificial Intelligence (e.g. ChatGPT)	
12. How often did you use the following **artificial intelligence tools** to prepare for the MLA AKT exam?	MCQ-LS
13. How often did you use these AI tools for the following learning tasks?	MCQ-LS
Other Resources (e.g. YouTube, Study Notes…)	
14. How often did you use the following **other resources** to prepare for the MLA AKT exam?	MCQ-LS
15. Can you explain in a few words why any of the resources you ticked for the previous question, helped with your MLA preparation?	Free Text
Resources for preparing for the Clinical and Professional Skills Assessment (CPSA) of the MLA	
16. How often are you using the following resources to **specifically prepare for the Clinical and Professional Skills Assessment (CPSA) of the MLA**?	MCQ-LS
17. Can you explain in a few words why any of the resources you ticked for the previous question, are helping you for your CPSA preparation?	Free Text
18. Please estimate what percentage of your time preparing for your MLA (AKT and CPSA) did you use **KMMS produced resources** compared to **external resources** (like books, question banks, flash cards…? )	0–100% Slider

**Multiple-Choice Questions Likert scale (MCQLS)** = students were able to answer “Never”, “Sometimes”, “Often” or “All the time”. **Multiple-Choice Questions Predefined Answer Responses (MCQPAR)** = students were given a set of predefined answers to tick. **Free Text** = students were able to answer freely. **Slider** = students were able to choose their percentage answer on a sliding scale.

Eight different types of learning resources were included in the survey: Books, Question Banks, Flashcards, Learning Platforms, Clinical Decision Support Tools, Generative AI Tools, Other Resources and CPSA Support Tools. Under “Learning Platforms”, we subsumed resources which provide bite-sized curriculum content using different media like text, animation or video. The “Other Resources” group included learning resources that did not fit into any of the other groups, such as self-curated personal study notes, Medical School Council (MSC) practise papers and anatomy resources or journals.

### Resources Included in the eSurvey

In total, 55 resources (49 for the AKT exam and 6 for the CPSA) were added to the groupings, based on recommendations from published literature, medical school provided resources, and from feedback and suggestions collated from KMMS academics and students (Table 2). Some resources such as *Geeky Medics* or *Zero to Finals* were allocated to more than one group because these platforms provided multiple types of resource. For clarity, these different allocations were counted as separate resources. Geeky Medics was divided into: *Geeky Medics – Question Bank*,* Geeky Medics – Flashcards and Geeky Medics-Learning Platform*. Zero to Finals was separated into: *Zero to Finals – Flashcards* and *Zero to Finals – Learning Platform.*

**Table 2:** Resources included in eSurvey

Resources included in the eSurvey	Type
1. Kumar and Clark’s Clinical Medicine [19] 2. Titles from the “Oxford Handbook” series (e.g. Oxford Handbook of Clinical Medicine, Oxford University Press) 3. Psychiatry[20] 4. Essential Primary Care [21] 5. Pass the PSA[22] 6. Medical Ethics [23] 7. Titles from the “Crash Course” series (e.g. Crash Course in General Medicine, Elsevier) 8. Titles from the “At a Glance” series (e.g. Medicine at a Glance, Wiley) 9. 100 Cases in General Practice [24] 10. Medicine in a Day [25] 11. The ECG Made Easy [26]	**Books**
12.PassMedicine 13. Clinical Key Student MLA Mock Exams 14. Clinical Key Student Question Bank 15. Geeky Medics – Question Bank 16. Quesmed 17. OSCEstop 18. BMJ OnExamination 19. Pastest	**Question Banks**
20. Anki 21. Brainscape 22. Zero to Finals - Flashcards 23. Geeky Medics - Flashcards 24. Quizlet 25. KenHub	**Flashcards**
26. Osmosis 27. RX Bricks 28. SCRIPT 29. Capsule 30. Speaking Clinically 31. Geeky Medics – Learning Platform 32. Zero to Finals – Learning Platform 33. Sketchy	**Learning Platforms**
34. UpToDate 35. BMJ Best Practice 36. Dynamed	**Clinical Decision Support Tools**
37. Copilot (Microsoft, Edge) 38. Gemini (Google, Chrome) 39. ChatGPT 40. ScopusAI 41. Apple Intelligence	**Artificial Intelligence Tools**
42. Self-curated personal study notes 43. Study notes curated by a peer 44. Medical School Council (MSC) practise papers 45. Anatomy resources (E.G., Complete Anatomy, Grey’s Student Anatomy [27]) 46. YouTube an Internet video resources 47. Podcasts and social media 48. Websites from health organisations EG NHS, NICE, General Medical Council, medical societies 49. Journal articles	**Other Resources**
50. Macleod’s Clinical Examination [28] 51. Clinical Skills Net 52. OSCEstop 53. OSCE part of PassMedicine 54. TBL content on KMMS learn (the medical school virtual learning environment) 55. Clinical Skills Hub on KMMS learn (the medical school virtual learning environment)	**CPSA Support Tools**

### Analysis

MCQ-LS responses were individually weighted for ranking purposes. A weight (w) was assigned to each Likert scale option as follows:

- Never: w₁ = 1.
- Sometimes: w₂ = 2.
- Often: w₃ = 3.
- All the time: w₄ = 4.

The frequency of responses for each option (n₁, n₂, n₃, and n₄) was multiplied by the corresponding weight, and the results were summed to calculate the weighted score:

$$\:\mathbf{W}\mathbf{e}\mathbf{i}\mathbf{g}\mathbf{h}\mathbf{t}\mathbf{e}\mathbf{d}\:\mathbf{S}\mathbf{c}\mathbf{o}\mathbf{r}\mathbf{e}\:=\:\left({\mathbf{n}}_{1}\:\times\:{\mathbf{w}}_{1}\right)+\:\left({\mathbf{n}}_{2}\:\times\:\:{\mathbf{w}}_{2}\right)+\:\left({\mathbf{n}}_{3}\:\times\:{\mathbf{w}}_{3}\right)+\:\left({\mathbf{n}}_{4}\:\times\:{\mathbf{w}}_{4}\right).$$

The MCQ-PAR responses were collated and presented descriptively, along with free text data, which was not extensive, so did not necessitate thematic analysis. Correlation between demographic data and frequency of use of different resources were calculated by Fisher’s Exact test. The final question in which students estimated the percentage of time they used medical school produced resources, compared to external resources, is presented descriptively.

## Results

### Demographics

A total of 31 out of 64 students of the medical school completed the eSurvey, giving a response rate of 48%. Three students started the survey but did not complete it and were excluded from analysis. Among respondents, 48% (*n* = 15) were male and 52% (*n* = 16) were female; 81% (*n* = 25) were aged 21–24 years, 16% (*n* = 5) aged 25–29 years, and 3% (*n* = 1) above 30 years old. The highest level of study for 48% (*n* = 15) of participants was A-level, followed by 35% (*n* = 11) who have BSc/BA degrees, 3% (*n* = 1) with a master’s degree, 3% (*n* = 1) with a PhD and 11% (*n* = 3) with “Other” qualifications (e.g., international qualifications). Only one student was a registered health professional.

### Ranking of Resources Students Used To Prepare for the UKMLA-AKT

The 49 resources were rank ordered, as used by students to prepare for the UKMLA-AKT (Table 3), with the question bank resource *PassMedicine* achieving the highest weighted score of 121. All students used this resource, with 90% (*n* = 28) using it “All the Time” and 10% (*n* = 3) using it “Often”. *Passmedicine* far outweighed Geeky Medics-Question Bank, Self-Curated Personal Study Notes and Zero to Finals-Learning Platform, which scored 85, 83 and 83 respectively. It should be noted that this study did not look at combinations of resources and therefore which resource may better complement another.

**Table 3:** Ranking of resources used for UKMLA - AKT Preparation by weighted score

Rank	KMMS provided/purchased	Title	Weighted Score	Resource Type
1	Yes	PassMedicine	121	Question Bank
2	No	Geeky Medics – Question Bank	85	Question Bank
3	No	Self-Curated Personal Study Notes	83	Other Resources
3	No	Geeky Medics – Learning Platform	83	Learning Platform
3	No	Zero to Finals – Learning Platform	83	Learning Platform
6	No	Anki	80	Flashcards
6	No	Medical School Council (MSC) Practice Papers	80	Other Resources
8	No	YouTube and Internet Video Resources	70	Other Resources
9	Yes	Pass the PSA	69	Book
10	No	ChatGPT	68	GenAI
11	Yes	Osmosis	67	Learning Platform
12	No	Zero to Finals - Flashcards	60	Flashcards
13	No	Websites from Health Organisations (e.g. NHS, NICE, General Medical Council, Medical Societies)	59	Other Resources
14	Yes	The ECG Made Easy	58	Book
14	No	Quesmed	58	Question Bank
16	No	Podcasts and Social Media	56	Other Resources
17	No	Geeky Medics - Flashcards	54	Flashcards
18	Yes	Kumar and Clark	52	Book
19	No	BMJ Best Practice	51	Clinical Decision Support Tools
20	Yes	Oxford Handbooks	49	Book
20	Yes	Crash Course series	49	Book
22	Yes	Psychiatry	48	Book
23	Yes	Capsule	46	Learning Platform
24	No	Study Notes curated by a Peer	44	Other Resources
25	Yes	Medicine in a Day	42	Book
26	Yes	Clinical Key Student MLA Mock Exams	41	Question Bank
26	No	Pastest	41	Question Bank
28	Yes	Journal articles	40	Other Resources
29	Yes	RX Bricks	39	Learning Platform
29	No	OSCEstop	39	Question Bank
31	Yes	At a Glance series	38	Book
31	Yes	SCRIPT	38	Learning Platform
31	Yes	Anatomy Resources	38	Other Resources
34	No	Quizlet	37	Flashcards
34	No	UpToDate	37	Clinical Decision Support Tools
36	Yes	Essential Primary Care	35	Book
36	No	Brainscape	35	Flashcards
36	No	Gemini (Google, Chrome)	35	GenAI
39	Yes	Clinical Key Student Question Bank	34	Question Bank
40	Yes	100 Cases in General Practice	33	Book
41	Yes	Medical Ethics	32	Book
41	No	KenHub	32	Flashcards
41	No	Dynamed	32	Clinical Decision Support Tools
44	Yes	Speaking Clinically	31	Learning Platform
44	No	BMJ OnExamination	31	Question Bank
44	No	Sketchy	31	Learning Platform
44	No	Copilot (Microsoft, Edge)	31	GenAI
48	Yes	ScopusAI	29	GenAI
49	No	Apple Intelligence	28	GenAI

Of the total number of resources used by students (*n* = 49), 45% (*n* = 22) were purchased/provided by the medical school and its libraries. 55% (*n* = 27) were either free resources or purchased by the students. The mean weighted score for purchased/provided resources was 47, compared to 53 for those resources which were not currently purchased/provided by the medical school or its libraries.

### Books

Students were asked about their use of traditional textbooks such as *Kumar and Clark’s Clinical Medicine* or the *Oxford Handbooks series*, and about high yield review books (some of which focussed their newest editions on UKMLA preparation) such as the *Crash Course series*. All of the books listed in the survey were core titles from the Y5 reading lists. The average weighted score for books was 46. *Pass the PSA*, a title preparing students for the mandatory Prescribing Safety Assessment scored highest (Table 4).

**Table 4:** Frequency and ranking of books students used for UKMLA - AKT Preparation

Rank	Resource	Never	Sometimes	Often	All the time	Weighted Score
1	Pass the PSA	11	7	8	5	69
2	The ECG Made Easy	9	17	5	0	58
3	Kumar and Clark Clinical Medicine	14	14	2	1	52
4	Oxford Handbooks	16	12	3	0	49
4	Crash Course Series	17	11	2	1	49
6	Psychiatry	17	11	3	0	48
7	Medicine in a Day	24	5	0	2	42
8	At a Glance series	26	3	2	0	38
9	Essential Primary Care	27	4	0	0	35
10	100 Cases in General Practice	29	2	0	0	33
11	Medical Ethics	30	1	0	0	32
					Average	46

In the open answer field of the MCQ-LS questions, students were asked about additional books used for preparation, which were not listed. The following titles were mentioned: *Essential Surgery* [29] *Grant’s Atlas of Anatomy* [30] ,*Mind Maps for Medicine* [31], *The Geeky Medics OSCE Revision Guide* [32] and *The Zero to Finals* textbooks series [33]. Two students explicitly stated that they did not use any books for their UKMLA preparation.

**Fig. 1:**
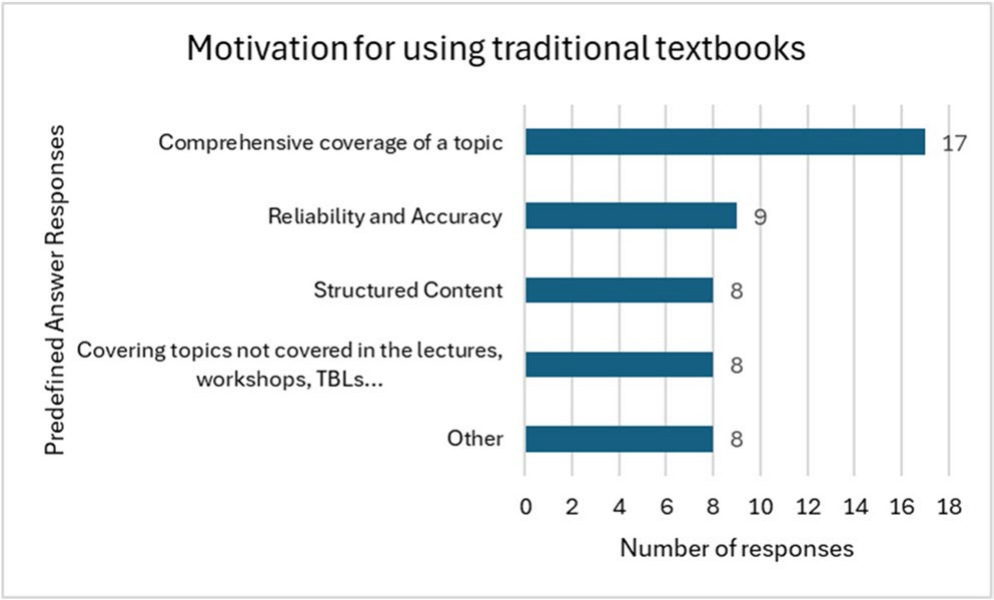
Motivation for using traditional textbooks

Specifically asked in the MCQ-PAR question about their reasons for using traditional textbooks rather than exam preparation books, 55% (*n* = 17) students selected “comprehensive coverage of a topic”, followed by “reliability and accuracy” 29% (*n* = 9) (Fig. 1).

In the open answer question (“Other”), one student explained that a textbook “*gives an understanding of a topic from start to finish instead of random bullet points*. *If you’ve read it*,* you know the topic*.” However, 13% (*n* = 4) students stated that they either did not use textbooks or found them unhelpful.

### Question Banks

*Passmedicine* was the highest ranked and most frequently used resource for UKMLA-AKT preparation, followed by *Geeky Medics* (Table 5). No other question banks other than the ones already listed were mentioned by students.

**Table 5:** Frequency and ranking of question banks students used for UKMLA - AKT Preparation

Rank	Resource	Never	Sometimes	Often	All the time	Weighted Score
1	PassMedicine	0	0	3	28	121
2	Geeky Medics	0	12	15	4	85
3	Quesmed	15	7	7	2	58
4	Clinical Key Student MLA Mock Exams	25	3	2	1	41
4	Pastest	23	6	2	0	41
6	OSCEstop	24	4	1	1	39
7	Clinical Key Student Question Bank	27	2	1	0	34
8	BMJ OnExamination	29	1	0	0	31
					Average	56

**Fig. 2:**
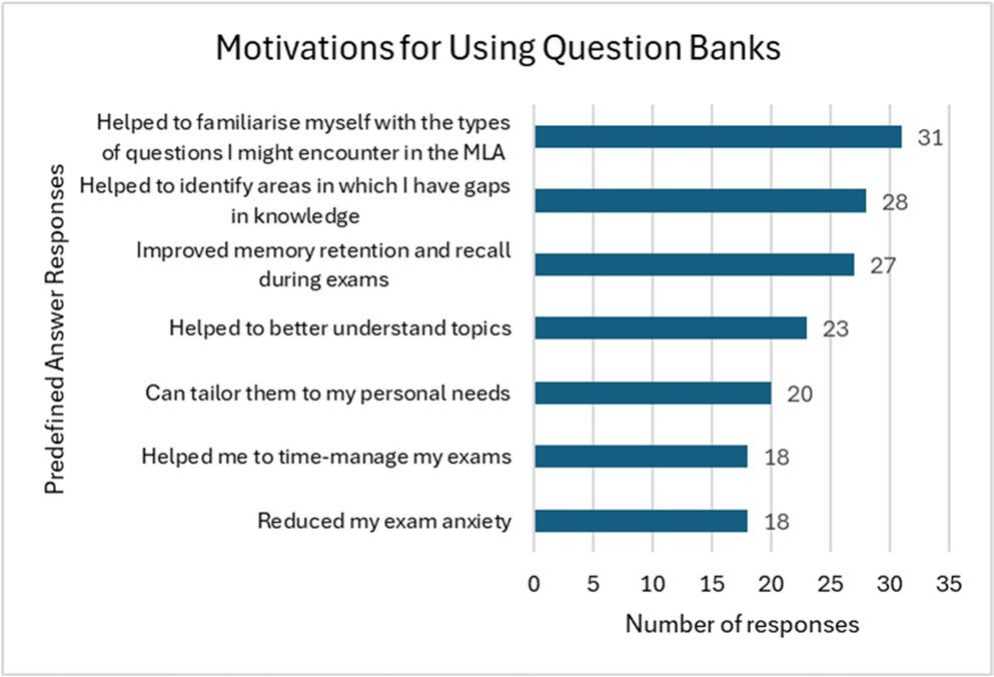
Motivation for using question banks

In the MCQ-PAR question, familiarising themselves with MLA question types, identifying gaps in knowledge and memory retention were the main reasons why Question Banks were used (Fig. 2).

### Flashcards

The most frequently used flashcards were *Anki*, closely followed by *Zero to Finals* and *Geeky Medics* (Table 6).

**Table 6:** Frequency and ranking of flashcards students used for UKMLA - AKT Preparation

Rank	Resource	Never	Sometimes	Often	All the time	Weighted Score
1	Anki	8	5	10	8	80
2	Zero to Finals	15	6	7	3	60
3	Geeky Medics	15	10	5	1	54
4	Quizlet	27	2	2	0	37
5	Brainscape	28	2	1	0	35
6	KenHub	30	1	0	0	32
					Average	50

**Fig. 3:**
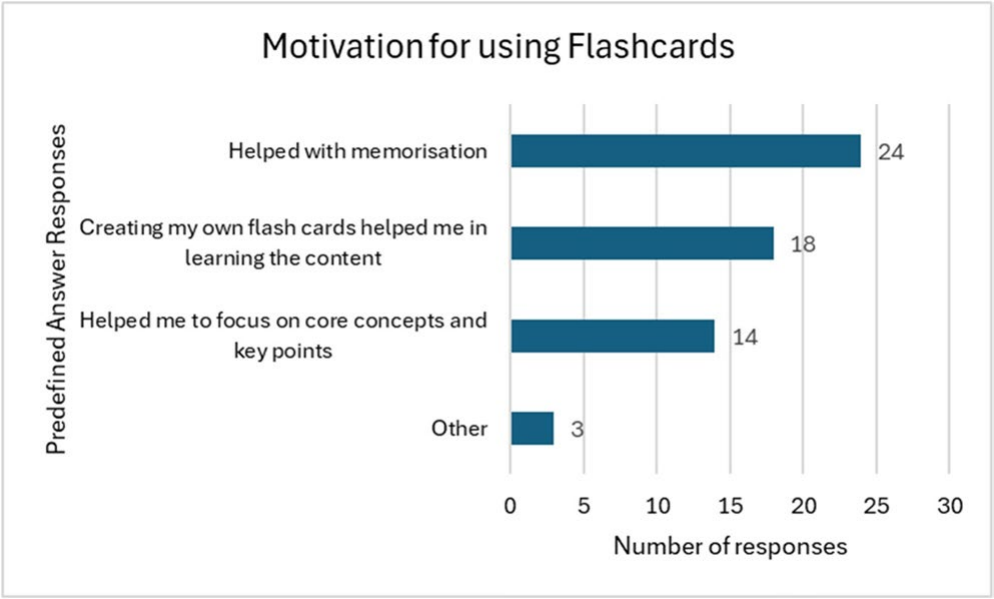
Motivation for using flashcards

The main motivation for using flashcards was help with recall and memorisation (Fig. 3). In the open answer question, one student highlighted the importance of spaces repetition by stating that “*Spaced repetition is the most effective and proven method. I made my own cards which took time*,* but Anki is pretty much all I did*,* and I just did a little each day*,* and it served me much better than when I’ve ever taken notes.’’*.

### Learning Platforms

Third-party resources, not provided by the medical school, such as *Geeky Medics* and *Zero to Finals* were the most frequently used by students (Table 7).

**Table 7:** Frequency and ranking of learning platforms students used for UKMLA - AKT Preparation

Rank	Resource	Never	Sometimes	Often	All the time	Weighted Score
1	Geeky Medics	2	10	15	4	83
1	Zero to Finals	5	8	10	8	83
3	Osmosis	6	16	7	2	67
4	Capsule	17	13	1	0	46
5	RX Bricks	25	5	0	1	39
6	SCRIPT	24	7	0	0	38
7	Speaking Clinically	31	0	0	0	31
7	Sketchy	31	0	0	0	31
					Average	52

The MCQ-PAR questions showed that students mainly used resources such as *Osmosis* or *RX Bricks* to revise pre-clinical curriculum content Reasons students used learning platforms were:

- *Good structure easy to understand and reinforce content*.
- *Learning content not covered/covered poorly during lectures*.
- *Concise explanations and breakdown of conditions*.
- *Good substitute for big textbooks.*

### Clinical Decision Support Tools

**Fig. 4:**
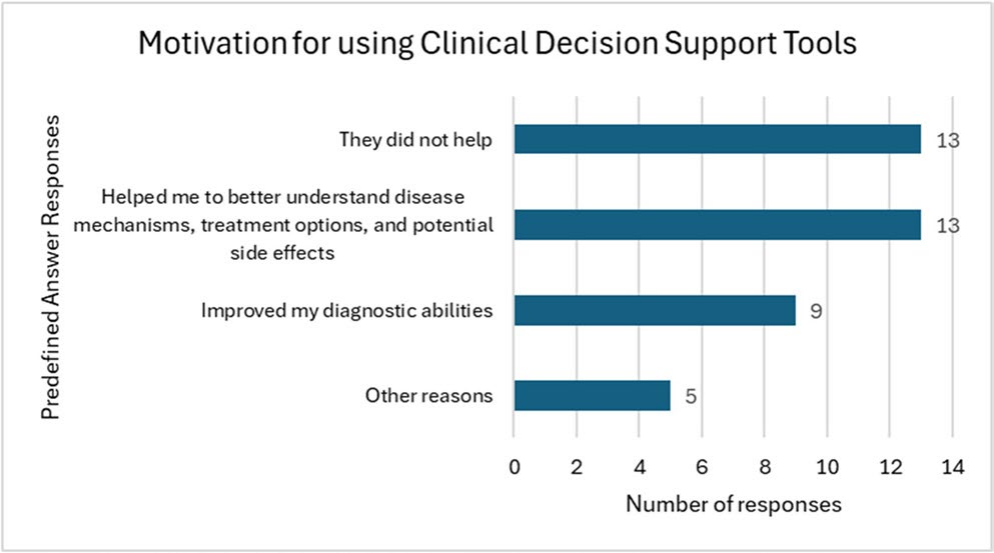
Motivation for using Clinical Decision Support Tools

Clinical Decision Support Tools such as *BMJ Best Practice*,* Up-to-Date and Dynamed* are provided to KMMS students whilst they are on longitudinal placement in NHS Trusts (Table 8). When asked about their motivation for using these tools, an equal proportion of students mentioned that they were either *not helpful* at all (42%, *n* = 13), or, that they *helped them to better understand disease mechanisms*,* treatment options*,* and potential side effects* (45%, *n* = 13) (Fig. 4).

**Table 8:** Frequency and ranking of clinical decision support tools students used for UKMLA - AKT Preparation

Rank	Resource	Never	Sometimes	Often	All the time	Weighted Score
1	BMJ Best Practice	18	7	5	1	51
2	UpToDate	27	2	2	0	37
3	Dynamed	30	1	0	0	32
					Average	40

### Generative AI Tools

*ChatGPT* was the most frequently used GenAI tool, used by 68% (*n* = 21) of students. *Gemini* and *CoPilot* were scarcely used, and *ScopusAI* (a tool provided by the medical school) and *Apple Intelligence* were not used at all (Table 9). Students who did use GenAI, mainly used it to explain medical concepts and conditions (Table 10).

**Table 9:** Frequency and ranking of generative AI tools students used for UKMLA - AKT Preparation

Rank	Resource	Never	Sometimes	Often	All the time	Weighted Score
1	ChatGPT	10	10	6	5	68
2	Gemini (Google, Chrome)	26	3	1	0	35
3	Copilot (Microsoft, Edge)	29	1	0	0	31
4	ScopusAI	29	0	0	0	29
5	Apple Intelligence	28	0	0	0	28
					Average	38

**Table 10:** Motivation for using generative AI tools

Rank	Resource	Never	Sometimes	Often	All the Time	Weighted Score
1	Explaining medical concepts and conditions	9	14	3	5	66
2	Creating memory aids and mnemonics	14	9	4	4	60
3	Generating differential diagnoses	16	6	3	5	57
4	Practicing for OSCEs by creating virtual patients	24	4	4	2	52
5	Writing a medical question for revision	19	8	1	3	50

In the open answer question, one student, who also stated that they use ChatGPT *all the time*, provided insight on how they use ChatGPT: “*I would put questions I got wrong from Passmed on ChatGPT and get it to explain to me why the answer was this or if I wanted more detail of an explanation from the passed questions I would ask ChatGPT to teach me this for the standard of a UK final year med student.”*

### Other Resources

Amongst “Other Resources” (Table 11), self-curated study notes and Medical School Council (MSC) practice papers ranked most highly.

**Table 11:** Frequency and ranking of “Other Resources” students used for UKMLA - AKT Preparation

Rank	Resources	Never	Sometimes	Often	All the Time	Weighted Score
1	Self-Curated Personal Study Notes	4	9	11	7	83
2	Medical School Council (MSC) Practice Papers	2	16	6	7	80
3	YouTube and Internet Video Resources	4	13	12	1	70
4	Websites from Health Organisations (e.g. NHS, NICE, General Medical Council, Medical Societies)	13	8	6	3	59
5	Podcasts and Social Media	13	13	3	2	56
6	Study Notes curated by a Peer	19	8	3	0	44
7	Journal articles	23	5	1	1	40
8	Anatomy Resources	22	8	0	0	38
					Average	59

In the open answer question, students provided feedback on their motivation for using personal study notes and internet resources only:

#### Self-Curated Personal Study Notes

The ability to easily share these with peers or to structure notes in a students’ own way were mentioned by four students. One student highlighted their personal study style in more detail:

“*I have made my own written notes since year 3*,* this is colour coded with pictures which helps me visually recognise conditions and helps me easily picture my notes when doing an exam question*,* almost like spatial recognition. My notes would be a collation of kmms lecture notes*,* passmed high yield textbook*,* zero to finals notes and osmosis”’.*

#### YouTube and Internet Video Resources

Eight students provided some comments on this learning resource:

- YouTube lectures are engaging.
- Learning with videos is easier for them than to read lots of pages in textbooks.
- YouTube videos and podcasts helped to better understand concepts.

Overall, some generalised feedback included:

- *“The Zero to Finals book literally had everything in one place*,* alongside Passmedicine to supplement knowledge of guidelines and then the MLA created mocks just to understand the writing style of the MLA writers and what they mean when they’re asking a question”*.
- *“Passmedicine*,* Anki personal notes and Zero to Finals all helped to organise and consolidate medical knowledge”*.
- *“Passmedicine was most important for question practice”*.
- *“Lots of MLA AKT questions based on NICE guidelines so useful to understand some of the guidelines e.g. asthma management in more details”*.
- *“AI was useful as it’s basically like having your own tutor”.*

### Ranking of Resources Students Used for UKMLA - CPSA Preparation

Resources used by students to prepare for the UKMLA-CPSA were also weighted and rank ordered, with the resources *Clinical Skills Net* and *Clinical Skills Resources on the medical school Virtual Learning Environment (VLE)* achieving the highest weighted scores of 64 (Table 12). Out of the 6 resources listed, 66% (*n* = 4) (were provided by the medical school and 24% (*n* = 2) were not provided.

**Table 12:** Resources for UKMLA clinical and professional skill assessment (CPSA) Preparation

Rank	KMMS provided resource	Resource Title	Weighted Score
1	No	Clinical Skills Net	64
1	Yes	Clinical Skills Hub on VLE	64
3	No	OSCEstop	47
4	Yes	Macleod’s Clinical Examination (Dover, 2024)	45
5	Yes	OSCE Part of PassMedicine	44
6	Yes	TBL content on KMMS Learn	36

In the open answer question, *Geeky Medics was* frequently mentioned as an important resource for OSCE preparation. Many students highlighted its usefulness for practicing scenarios, accessing mark schemes, and providing a wealth of OSCE stations. Some students also noted the availability of a subscription for more practice.

Students mentioned that they use *Clinical Skills.net* for its comprehensive mark schemes and as an alternative to the KMMS checklists, which some found less effective.

KMMS-specific resources, such as examination notes and checklists are also heavily used. Students appreciate that these materials are tailored to KMMS assessment practices, helping them ensure they perform steps correctly and are less likely to be penalised for their examination style or procedure. The relevance of these resources to KMMS-style OSCEs and their provision of patient scenarios similar to assessments were also cited as beneficial.

Overall, students leverage these tools to understand potential exam topics, practice their clinical skills and to prepare for specific scenarios.

### KMMS in-house Resources vs. External Resources

Overall, students estimated that they spent 20% of their time engaging with KMMS in-house resources compared to 80% engagement with external resources, for both the AKT and the CPSA part of the UKMLA. Only 13% (*n* = 4) students used KMMS in-house resources the same or more than external resources. The 20% engagement with in-house resources is likely attributable to time spent on CPSA exam preparation only, given that KMMS in-house resources on the VLE were ranked equally with Clinical Skills Net, yet no in-house resources were ranked for the AKT preparation.

### Correlations between Frequency of Use and Demographics

There were significant correlations between gender, age and level of education, with frequency of use of resources. Only one book, *Essential Primary Care (Blythe*,* 2017)*, showed significant associations with “Age group” (*p*=.016) and “Level of study” (*p* = .010); 96% (*n* = 24) from the 21–24 age group versus 60% (*n* = 3) from the 25–29 age group, never used the book, implying that the older age group used it more often. However, overall usage was low with 40% (*n* = 2) from the 25–29 age group and a single student from each of the 21–24 and the 30 + age groups, using it at least “sometimes”. The more frequent use by the 25–29 age group correlates with “Level of study”, where 4 students with a degree used it at least “sometimes” compared to none of the A-Level students.

Question bank, *Pastest*, was significantly associated with “Level of Study” where 31% (*n* = 5) post grad students used it “sometimes” compared to only 8% (*n* = 1) students with A-Level education. The *Brainscape and Geeky Medics Flashcards* were significantly associated with “Age Group” (*p*=.032; *p*=.041 respectively). *Brainscape* was used “sometimes” by 8% (*n* = 2) students from the 21–24 age group, and “often” by a single student in the 30 + age group. In contrast, *Geeky Medics* was used “often” by one student from each of the higher age groups (25–29 and 30+), yet “sometimes” by 40% (*n* = 10) or “often” by 16% (*n* = 4) of the A-Level 21–24 year olds (*p*=.025).

YouTube and Internet Videos showed a significant correlation with gender; 100% (*n* = 16) of female students used it either “often” or “sometimes” compared to 64% (*n* = 9) of male students, with 29% (*n* = 4) males never using it and one using it “All the time” (*p*=.043). Lastly, for the CPSA, 33% (*n* = 5) of A-Level students used *Macleod’s Clinical Examination (Dover*,* 2024)* “sometimes” compared to 53% (*n* = 7) of BSc/BA, MSc/MA and PhD students, who used it either “sometimes” or “often” (*p*=.047).

## Discussion

This case study provides valuable insights into Year 5 students’ resource preferences for UKMLA preparation, largely aligning with existing literature on high-stakes national licensing examinations like the USMLE. The pre-eminence of question banks, with *Passmedicine* leading the ranking in this study, directly supports findings by Burk-Rafel et al. [10], Wynter et al. [9] and Fisher et al. [11], who highlight their critical role in exam preparation and performance. Students’ main motivations for using question banks are the simulated exam experience and identifying knowledge gaps, which is also consistent with the literature [11]. The high usage of flashcards, particularly Anki, aligns with and reinforces prior research findings [3, 14, 15], suggesting these tools are pedagogically effective in promoting efficient, targeted studying.

Traditional textbooks like *Kumar & Clark*,* Macleod’s Clinical Examination* or titles from the *Oxford Handbook series*, despite being core texts and often promoted in lectures, were lower ranked than some of the “high-yield” exam preparation books such as *Pass the PSA* or *The ECG Made Easy*, thereby indicating a preference for concise, exam-focused exam preparation texts, rather than topic textbooks. Again, this trend is strongly supported by the literature showing the wide use of exam preparation books like ‘*First Aid for the USMLE Step 1*’ by medical students preparing for the USMLE [3, 13].

This case study also endorses the literature on the high usage of audiovisual learning resources for UKMLA preparation [3]; *Osmosis* (a video platform) ranked highly, along with *YouTube and Other Internet Video* resources. This frequent use of audiovisual resources may be related to individual learning style as some students mentioned in their open-ended responses that they find learning from videos easier than reading numerous pages in textbooks.

In this study, students were asked about their use of AI for preparing for the UKMLA. They demonstrated a preference for ChatGPT, likely influenced by its leading position among generative AI tools [34]. Students did not mention any other AI tools, perhaps indicating a lack of awareness of AI tools in general and in turn, indicating potential for more formal integration of GenAI in supporting medical students with preparation for national licensing exams. Kung et al. [35] stated that GenAI could support students with clinical reasoning and by surfacing new and non-obvious concepts. In fact, one student in this study commented that *AI is basically like having your own tutor*, signifying an emerging trend in personalised learning and exam preparation strategies, with AI having the potential to support students in various aspects such as explaining medical concepts, creating memory aids and mnemonics, generating differential diagnoses or bridging knowledge gaps.

This case study also highlights the use of clinical decision support tools such as *Up-To-Date*,* BMJ Best Practice* and *Dynamed* for UKMLA preparation. Students have access to these resources whilst on their clinical placements and this study indicates that for some students they play an important role in their exam preparation. They help them to better understand disease mechanisms, treatment options, and potential side effects and were used to improve their diagnostic abilities. *BMJ Best Practice* emerged as the most used resource, perhaps due to its availability in all trust libraries compared to *Up-To-Date* or *Dynamed.*

Regarding preparation for the CPSA component of the UKMLA, this study shows good uptake of resources produced by the medical school, which emphasises the value in providing these internal, tailored, materials for practical assessments, in addition to external resources such as *Clinical Skills Net*. This highlights the importance of academic guidance and the provision of high-quality, curriculum-aligned materials that ensure students are well-prepared using trusted, relevant resources. On the other hand, for the UKMLA-AKT, the majority of students preferred the third-party resources either provided by KMMS or purchased/accessed by themselves.

Existing literature clearly highlights the risk of financial burden and inequalities if inadequate resources are provided by a medical school, meaning students have to buy them for themselves [3, 10, 11, 18] and strongly advocates for medical schools to offer resources to their students to support their preparation [3, 11]. This case study provides evidence on the importance of third-party tools for UKMLA preparation and encourages medical schools and libraries to provide these resources to their students.

### Limitations

The small sample size within a single institution limits generalisability of the results, however, the clear alignment with current literature on preparation for national licensing exams such as the USMLE, indicates that these results could be relevant for other UK-based medical schools.

This study did not correlate specific resource usage with individual student performance outcomes, nor did it interrogate usefulness of specific bundles of resources. But it is worth noting the 100% pass rate of the Y5 cohort who took part in this study; passing both parts of the UKMLA with a class average of 72% for the AKT part. The overall success of the cohort provides contextual supplementary evidence that the resources students used, supported a successful UKMLA preparation.

## Future Directions

Future research could enhance generalisability by employing larger, multi-institutional data across UK medical schools. Broader recruitment and inter-institutional collaboration may strengthen the applicability of findings to national UKMLA preparation. Additionally, studies examining the relationship between specific combinations of preparatory resources and individual performance in the AKT and CPSA may help identify more effective preparation strategies. Further qualitative research is needed to compare resource preferences and to explore additional factors such as perceived effectiveness, accessibility, cost, or alignment with learning styles. Such research would provide valuable contextual insight and complement the quantitative findings of the present study.

## Conclusion

This case study has demonstrated that medical students’ rely on third-party resources, particularly question banks, to prepare for the UKMLA, along with medical school produced (in-house) resources for CPSA preparation. The extensive use of third-party tools mirrors trends in comparable national licensing examinations such as the USMLE. Two books *Essential Primary Care (Blythe*,* 2017)* and *Macleod’s Clinical Examination (Dover*,* 2024)*, the question bank *Pastest* and the flashcard *Brainscape* were used more by mature students, whereas flashcards from *Geeky Medics* were used more by the younger students. In addition, more female than male students have use *YouTube and Internet Videos*. Notably, the results emphasise the emerging role of GenAI as a “personal tutor,” further shaping students’ study strategies for the UKMLA. Given the critical role of third-party tools and the potential financial burdens and inequalities for students who can’t afford them, medical schools are called upon to purchase these tools as this study provides preliminary evidence that they significantly contribute to success in UKMLA examinations.

## Data Availability

The data that support the findings of this study are available from the corresponding author upon reasonable request.

## Abbreviations

UKMLA: UK Medical Licensing Assessment
KMMS: Kent and Medway Medical School
AKT: Applied Knowledge Test
CPSA: Clinical and Professional Skill Assessment
Y5: Year 5; GAI (Generative Artificial Intelligence)
